# Genetic and Molecular Control of Somatic Embryogenesis

**DOI:** 10.3390/plants10071467

**Published:** 2021-07-17

**Authors:** Camille Salaün, Loïc Lepiniec, Bertrand Dubreucq

**Affiliations:** Institut Jean-Pierre Bourgin, INRAE, AgroParisTech, Université Paris-Saclay, 78000 Versailles, France; camille.salaun@inrae.fr (C.S.); loic.lepiniec@inrae.fr (L.L.)

**Keywords:** embryogenesis, LAFL, transcriptional regulation, epigenetic control

## Abstract

Somatic embryogenesis is a method of asexual reproduction that can occur naturally in various plant species and is widely used for clonal propagation, transformation and regeneration of different crops. Somatic embryogenesis shares some developmental and physiological similarities with zygotic embryogenesis as it involves common actors of hormonal, transcriptional, developmental and epigenetic controls. Here, we provide an overview of the main signaling pathways involved in the induction and regulation of somatic embryogenesis with a focus on the master regulators of seed development, LEAFY COTYLEDON 1 and 2, ABSCISIC ACID INSENSITIVE 3 and FUSCA 3 transcription factors whose precise role during both zygotic and somatic embryogenesis remains to be fully elucidated.

## 1. Introduction

Plants can propagate through sexual or asexual reproduction. In Angiosperms, zygotic or sexual reproduction is the most common and consists of the double fertilization of a female gametophyte (that contains the egg and the central cell) by the two sperm cells of a male gametophyte (pollen). Sexual reproduction leads to the formation of a seed that contains all the components required for seedling development: the differentiated zygotic embryo which will give rise to the future plant, storage compounds located in the cotyledons, endosperm and/or nucellus depending on the species, all surrounded by a protective tissue, the testa (formed by ovary integuments) [1]. Through meiosis and crosses, zygotic reproduction allows genetic diversity in the progeny and its propagation through seeds, that can be transported through wind, water (hydrochory) or animals (zoochory) [2]. However, plants are also able to reproduce in an asexual way, i.e., without meiosis and fertilization. For example, apomixis consists in the formation of a seed derived from a diploid cell of the ovule and can occur naturally in some species [3]. Vegetative reproduction is also widespread and used in horticulture, with different techniques such as layering, cuttings, and grafting.

This study focuses on somatic embryogenesis (SE), a complex process of clonal propagation by which plants can form embryos without meiosis and fertilization. This process involves the totipotency of plant cells, i.e., their ability for dedifferentiation and differentiation in new cellular types. The new plant derived from a somatic embryo is thus genetically identical to the mother plant. SE can occur spontaneously or in response to specific environmental conditions in some species such as Kalanchoe [4,5]. 

Understanding the mechanisms controlling somatic embryogenesis and its regulation is a key issue in plant biology, since clonal propagation through SE is widely used for various plants of agronomic interest, such as *Coffea* spp. [6], *Pinus* spp. [7], *Theobroma cacao* L. [8] and other species, as it facilitates the clonal multiplication of elite genotypes in a quite fast and efficient way [9]. Moreover, SE is also largely used for genetic transformation protocols of these agronomical species, and for other crops such as rice, soybean, maize, or wheat [10,11,12]. Because the efficiency of transformation is correlated with the embryogenic and regeneration capacity, comprehension of SE processes is of great importance.

However, somatic embryogenesis involves complex, or even not characterized, genetic inheritance [13]. Some species or specific genotypes can display a recalcitrance to SE and are unable to undergo it even under favorable conditions. Lastly, although somatic and zygotic embryogenesis (ZE) share some similar hormonal, transcriptional or epigenetic controls [14], they also display some specificities. In this review, the known regulatory mechanisms involved in SE and ZE are compared with a specific focus on the *LAFL* (*LEC1*, *ABI3*, *FUS3* and *LEC2*) gene regulatory network.

## 2. Onset of Somatic Embryogenesis

Somatic embryogenesis relies on the totipotency of plant cells, i.e., their capacity to dedifferentiate and differentiate in a new cell type [15,16]. Although some plant species such as *Kalanchoe daigremontiana* undergo spontaneous SE [4], this process is usually induced in vitro by a stress over the plant tissues. This stress, that is essential for SE induction, can take different forms (reviewed in [17]): a high level of plant hormones in the culture media is the most commonly used [18,19], but it also can be wounding, extreme pH, osmotic or heat shock, or treatments with different chemicals. The first stages of SE are characterized by the induction of the expression of numerous stress-related genes and especially those encoding transcription factors belonging to AP2/ERF, MYB, AUX/IAA, B3 or WUS/WOX families [19,20,21]. 

Somatic embryos can be induced directly from in vitro cultivated plant tissues (for example immature zygotic embryos) on a low-auxin medium. Alternatively, indirect SE can be induced by cultivating embryogenic tissues, such as callus, on an auxin-rich medium leading to the transition to somatic embryos by switching to a low-auxin medium [22,23]. The protocols used for industrial clonal reproduction or for the research on somatic embryos are usually based on indirect SE, since it allows the production of a large number of somatic embryos [22,23,24] and because direct SE can be ineffective for some species or genotypes, such as *Gossypium hirsutum* [25], *Capsicum chinense* Jacq., or *Cocos nucifera* L. [26].

## 3. Hormonal Control of Somatic Embryogenesis

Multiple events in the plant life cycle, such as development or responses to biotic and abiotic stresses, are controlled or regulated by hormones. Auxins are known to play essential roles in all the aspects related to plant development, from cellular division to elongation, through cell identity and organogenesis [27,28]. Auxin polar transport by the proteins PIN (efflux) and AUX (influx) leads to differential distributions, with gradients and maxima that are essential for plant development [29]. During zygotic embryo development, local auxin production, polar transport and gradient are mainly mediated by PIN proteins [30]. More specifically, in Arabidopsis, the expression of *PIN1* and *PIN7* genes in the apical part of the young embryo is at the basis of the acquisition of the apical-basal orientation [30,31], in concert with *AUX1*, *LAX1* and *LAX2*, playing a cooperative role [32].

Not surprisingly, auxin was thus shown to be a central player in the induction of SE as reviewed in [33]. Briefly, auxins and more specifically 2, 4-dichlorophenoxyacetic acid (2, 4-D), a synthetic auxin-like plant growth regulator, are largely used for inducing SE [19]. The treatment of plant tissues with 2, 4-D involves both a stress and the auxin response, biosynthesis and signaling pathway, as well as a transcriptional reprogramming and chromatin remodeling (as described below) [33]. Similar to zygotic embryogenesis, a polar transport and auxin gradient are necessary for the formation of a somatic embryo [34]. SE induction, as well as organogenesis and plant regeneration, depends on the addition of plant growth regulators (PGR) such as auxins and cytokinins [18]. Although many protocols adapted to different species and genotypes are available, a key element for embryo formation is the large variation in PGR concentration added to the culture media [24]. A majority of protocols are based on the use of auxins, alone or combined with cytokinins [24]. During SE induction, auxin response factor (*ARF*) genes display specific expressions being up- or down-regulated, suggesting that auxin signaling is central in the process [35]. Moreover, *YUCCA* and *AUX*/*IAA* genes that are respectively involved in auxin biosynthesis and response, are transcriptionally regulated during SE, including by LAFL transcription factors [36,37].

Growing plant tissues in vitro with high concentrations of auxin (2, 4-D), triggers a general reprogramming of somatic cell transcriptomes and modulates the expression of many SE-associated transcription factors [33]. Moreover, when protoplasts are cultured with a medium containing high concentrations of 2, 4-D, the size of their nuclei is significantly increased, suggesting a reorganization of the chromatin [38]. Finally, hormonal stress induces a modification of chromatin state, leading to the activation of transcription factors such as *WUS*, *LEC* genes or *BBM* that are specific to embryogenic programs [39]. These results suggest that hormones, especially auxin, trigger a general reprogramming of gene expression through chromatin modifications and activation of specific transcription factors.

## 4. Transcriptional Control of Somatic Embryogenesis 

The main way to regulate gene expression is the transcriptional initiation through transcription factors (TF). Transcriptional regulation performs an essential role in somatic embryogenesis [40]. In Arabidopsis, the ectopic expression of some transcription factors, such as *LEC* genes, *BBM*, *WUS*/*WOX* genes or *AGL15* can increase the efficiency of SE induction and lead to the formation of somatic embryos without adding hormones [23]. Mutations in these genes have a negative impact on the efficiency of SE induction [1,41,42]. Several of the TF are involved in hormonal signaling, while others control cellular differentiation and organogenesis. These transcription factors are described below.

### 4.1. LAFL (LEC1, LEC2, ABI3, FUS3)

**Figure 1:**
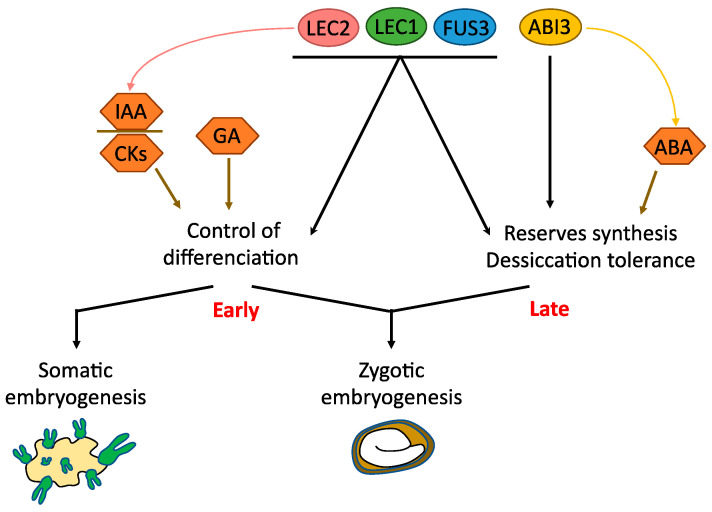
The roles of *LAFL* genes in somatic embryogenesis (SE) and zygotic embryogenesis (ZE). IAA: auxins, CKs: cytokinins, GA: gibberellins.

ABSCISIC ACID INSENSITIVE 3 (ABI3), FUSCA 3 (FUS3) and LEAFY COTYLEDON 2 (LEC2) are transcription factors forming the AFL group. They belong to the plant-specific B3 family of transcription factors, characterized by a highly conserved B3 DNA binding domain of ~110 amino-acid residues first characterized in ABI3/VP1 [43,44]. B3-containing factors can recognize specific target DNA with a 5’-*GATC*-3’ core sequence, known as *RY* boxes and conserved within the promoters of storage genes [45,46]. Together with LEAFY COTYLEDON 1 (LEC1), a NF-YB transcription factor [1] involved in CAAT box complexes [47], AFL form the LAFL group of master regulators of seed development [48,49,50]. They can genetically and/or physically interact altogether and form protein complexes to activate their target genes [46,51] and control various aspects of seed development, such as the accumulation of storage compounds or the acquisition of desiccation tolerance (Figure 1). The expression of LAFL genes is regulated by transcription factors, such as BBM [52], hormonal signaling (ABA, GA, IAA) [53] and chromatin modifications [52].

These genes are involved in somatic embryogenesis programs as well as in the initiation and maintaining of the embryogenic fate of plant cells [54] (Figure 1). Indeed, the ectopic expression of *LEC* genes (*LEC1*, *LEC2*, and *FUS3*) in Arabidopsis and in other species can induce the spontaneous formation of somatic embryos without addition of hormones in the culture medium [42,55,56,57,58,59]. The expression of *GhLEC1*, *GhLEC2* and *GhFUS3* is significantly higher in cotton (*Gossypium hirsutum*) genotypes permissive to callus differentiation, than in recalcitrant genotypes [60]. Consistent with this role, *lec1*, *lec2* and *fus3* simple and multiple mutations severely impair the ability to form somatic embryos in Arabidopsis [60].

**Figure 2:**
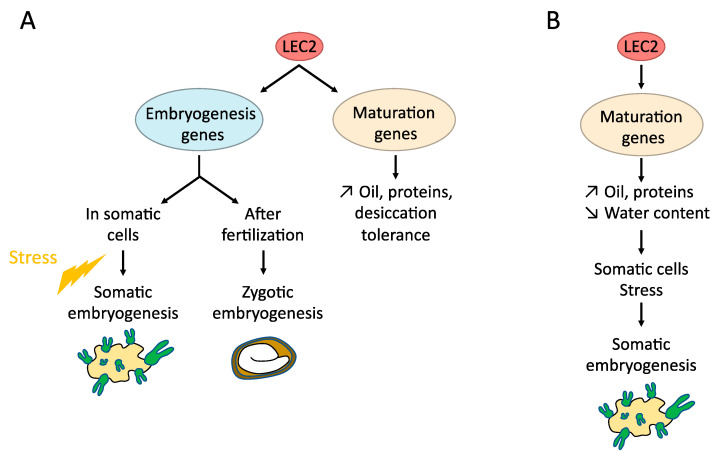
The possible roles of LEC2 in somatic embryogenesis. (**A**): LEC2 can have a dual function, one specific to embryogenesis, the other inducing maturation genes. The embryogenesis function may lead to SE or ZE depending on the type of cell it is expressed in, as well as possible stress and epigenetic modifications. (**B**): LEC2 induces stress through the accumulation of reserve specific compounds in the cells.

Several target genes of the LEC proteins can be directly involved in the regulation of somatic embryogenesis [33,61], such as *AGL15*, that is involved in hormonal signaling and controls the embryogenic induction [62]; *IAA30*, a main actor of auxin signaling and perception [63] or *LOB40,* a TF putatively involved in the formation of organ boundaries and gibberellins signaling [64]. LEC2 and AGL15 control each other in a feedback regulatory loop [33]. LEC2 is also known to rapidly induce the expression of auxin-related genes, such as *IAA1*, *IAA17* and *ACS4*, and key enzymes involved in auxin biosynthesis such as the *YUC* genes (*YUC1*, *YUC2*, *YUC4*, *YUC10*) [37,65]. However, among these auxin genes, only *YUC4* is a direct target of LEC2. Then, it can be hypothesized that LEC2 (and LEC1) can induce SE processes through different mechanisms (Figure 2). The LEC proteins activate the maturation program, leading to the increase in various storage compounds and the decrease in water content. These physiological changes would trigger a stress inducing SE competency and/or promotion of auxin activity in competent cells [65]. In addition, some genes usually expressed during zygotic embryo development and known to bring an embryogenic competence, such as *AGL15* for example, are directly induced by LEC2 [65]. Another hypothesis can be a dual function of LEC2, able to act as a regulator of seed maturation by regulating specific targets in complex with specific partners, and as a regulator of embryogenesis (both ZE and SE), by regulating other targets, in cooperation with other partners (Figure 2). 

ABI3 is involved in ABA-mediated developmental and metabolic processes, such as seed maturation, accumulation of seed proteins or the transition between embryo maturation and early seedling development [66]. ABI3 is essential for the emergence of roots from callus after induction of organogenesis. The *abi3*-6 mutant does not show any regeneration after passing from a hormone-rich to a hormone-free medium [67]. Nevertheless, the overexpression of *ABI3* does not induce somatic embryogenesis [68,69]. Because the expression of auxin-related genes is significantly weaker in the *abi3*-6 mutant, ABI3 can be involved in auxin distribution and/or homeostasis, with its role in ABA signal transduction and reserve accumulation, rather than in the initiation of SE as other LAFL. 

Although very similar in their B3 sequences and binding capabilities [46], LAFL are thus involved to a different extent in somatic embryogenesis (Figure 1 and Figure 2). This is consistent with their different DNA binding activities [46] and their specific expression patterns.

### 4.2. BABY BOOM (BBM)

BABY BOOM (BBM), also known as PLETHORA 4 (PLT4) is a transcription factor belonging to the AP2/ERF TF family characterized by a DNA binding domain of 70 amino-acid residues, that were first characterized in APETALA2 (AP2) and ethylene-responsive element binding proteins (EREBP) [70,71]. The AP2/ERF family is divided into two subfamilies: TFs that count one AP2/ERF DNA binding domain and are generally involved in response to abiotic and environmental stresses [72], and those counting two DNA binding domains, known to regulate developmental processes. BBM contains two AP2/ERF domains and is a member of the AINTEGUMENTA-LIKE (AIL) subfamily, composed of eight members, all carrying specific functions in the division of meristematic and embryonic cells and involved in the regulation of SE and its induction when they are ectopically expressed [33]. *BBM* is expressed in the zygotic embryo and regulates cell identity and the growth of the root meristem [73]. The ectopic expression of *BBM* in *Arabidopsis thaliana* and *Brassica napus* leads to the spontaneous formation of somatic embryos and cotyledon-like structures over plantlets [70]. However, other phenotypes are also induced by ectopic expression of *BBM* in these species, such as callus proliferation, ectopic shoot formation, alterations in leaf morphology and better regeneration of explants during in vitro culture, without adding hormones in the medium. BBM can thus have a stimulating role in cell proliferation and morphogenesis during both SE and ZE. BBM directly binds promoter regions of *LAFL* genes through its ANT/AIL binding motif and *AGL15* via another motif or an intermediate protein [52]. *LAFL* genes are upregulated after *BBM* induction, and together with AGL15, are *BBM*-positive regulators during SE processes and are key components of the BBM signaling pathway, although these interactions may be indirect. 

### 4.3. WUSCHEL (WUS) and WUSCHEL-RELATED HOMEOBOX (WOX) Genes

WUSCHEL (WUS) is a homeobox transcription factor that is described as a key regulator of meristematic stem cell fate [74]. It also performs an essential role in somatic embryogenesis, by promoting the vegetative-to-embryonic transition and the maintenance of embryogenic cell identity [75]. WUS is involved in the regulation of the embryogenic cell (totipotency) and meristematic cell (pluripotency) fates [76]. In Arabidopsis, *wus* mutants can form somatic embryos, although seedlings display the same phenotype as seed-derived *wus* plantlets, i.e., an absence of functional embryonic shoot apical meristem (SAM), and the typical *wuschel* stop-and-go development described in [74] consisting, among others, in the formation of new shoot meristems instead of organs at the primordia [77]. The overexpression of *WUS* and gain of function mutations can increase somatic embryo production or induce the formation of somatic embryos from vegetative tissues without addition of external hormones in *Arabidopsis thaliana* [75]. In many other species, such as *Gossypium hirsutum* [78] or *Coffea canephora* [79], the overexpression of *WUS* is also able to significantly increase the ability of a plant to form somatic embryos. In *Gossypium hirsutum*, the ectopic expression of *AtWUS*, increases the efficiency of callus differentiation of recalcitrant genotypes [60]. *WUS* can also be used as a marker gene of dedifferentiation after SE induction in *Medicago truncatula* [80]. During SE, *WUS* is upregulated and transcriptionally induces *LEC1*, *LEC2,* and *AGL15* genes [76]. Thus, we can hypothesize that SE originates from a cellular reprogramming followed by a process similar to ZE, at least in the signaling cascade of some key genes. When some master genes of ZE are knockdown (including *LAFL*), SE is impaired, favoring such a hypothesis. Two fully independent pathways exist, yielding to similar developmental structures. This hypothesis can be tested by comparing SE efficiency of the double mutants (e.g., *wus*/*lec2*) or double ectopic expressions to the single mutations/ectopic expression. 

The *WUSCHEL-RELATED HOMEOBOX* (*WOX*) genes share similar sequences with the WUS homeodomain and a specific WUS box located downstream of the homeodomain [81]. *WOX* genes are involved in the early embryonic patterning but also in different signaling pathways regulating several aspects of plant growth and induction of somatic embryogenesis [76]. Functions of the different known *WOX* genes in several species are reviewed in [76]. As an example, it was shown in *Medicago truncatula*, that *MtWOX9-1* overexpression improves SE efficiency, linked to an increase in AGL15 and AGL8 level of accumulation [82] and *WOX5* is significantly upregulated two days after SE induction and can serve, such as *WUS*, as a marker of dedifferentiation [80].

### 4.4. AGAMOUS-LIKE 15 (AGL15)

AGAMOUS-LIKE 15 (AGL15) is a transcription factor, belonging to the MADS domain protein family. These TFs are known to share a specific MADS DNA binding domain, containing a 55 to 60 amino-acid sequence conserved among eukaryotes [83]. *AGL* genes display diverse functions in plant development, more particularly in flower development, although AGL15 is mainly detected in developing embryos [84,85]. AGL15 can target and regulate the expression of numerous hormone-related genes, including gibberellic acid and ethylene metabolism but also auxin signaling via *GA2ox6* [86] and *IAA30* activation [62,86]. AGL15 activates its own expression [87] and regulates *LEC2*, *ABI3* and *FUS3* in a positive feedback regulatory loop [36]. The ectopic expression of *AGL15* in *Arabidopsis thaliana* and in soybean increases SE capacity [88,89]. While the efficiency of SE plant tissue can decrease with time, overexpression of *AGL15* maintains SE capacity for years, showing the role of AGL15 in the preservation of embryogenic capability [88]. The loss-of-function of AGL15 reduces the frequency of somatic embryo development [89]. 

### 4.5. WOUND INDUCED DEDIFFERENTIATION 1 (WIND1)

WOUND INDUCED DIFFERENTIATION 1 (WIND1) is an AP2/ERF transcription factor with two AP2 domains involved in callus formation in response to wounding and performs a critical role in the acquisition of regeneration competency and regulating cytokinin signaling [90,91]. The over- and ectopic expression of *WIND1* leads to the callus formation on explants without induction through the addition of hormones in the culture medium or wounding [91]. WIND1 is not directly involved in the induction of SE, but in the induction of callus formation and regeneration competency. However, the induction of *WIND1* expression followed by that of *LEC2* leads to the formation of callus within all the plant tissues that can be regenerated in a whole plant, showing that WIND1 acts upstream from LEC2 during SE and regeneration processes [92].

Through the example of several transcription factors, we have seen that transcriptional control performs a key role in the initiation and efficiency of somatic embryogenesis. Aside from their role in the transcriptional activation of their targets through the promoting regions, some eukaryotic TFs can activate their targets even if they are located in nucleosomal DNA that is less accessible. These specific TFs, called pioneer transcription factors, are usually submitted to strong (spatiotemporal) transcriptional or post-transcriptional controls and can act directly or indirectly by recruiting chromatin remodelers, thereby increasing local chromatin accessibility [93]. Pioneer factors are of primary importance in development since they allow the chromatin to be open and accessible for other factors to bind, and thus, are thought to enable cellular reprogramming [94]. This is the case for LEC1, that is a pioneer transcription factor capable of inducing chromatin modifications to different target genes: its overexpression leads to the spontaneous formation of somatic embryos on plantlets and its loss-of-function mutants are not able to do SE anymore [54,57,95,96,97].

## 5. Epigenetic Regulation of Somatic Embryogenesis

Although chromatin regulations are essential in developmental processes, relatively little data concerning the epigenetic regulation of somatic embryogenesis have been published (see [98] for review). DNA methylation profile and chromatin accessibility are constantly changing in response to cellular needs and environmental constraints [99]. The different abiotic stresses to which plants are subjected can either increase or reduce DNA methylation, depending on the type of stress (i.e., heavy metals, salt, temperature, culture density), the plant species or even the organ considered [100,101]. It should be noted that epigenetic changes induced can be transient or stable over a period of time, and can also be transmitted to the offspring [102]. Then, it can easily be hypothesized that epigenetic modifications correlate with SE processes [98]. In particular, the efficiency of cellular differentiation is linked to the methylation profile of DNA. The cellular reprogramming is often concomitant with significant changes in the chromatin status: not only DNA methylation but also histone modifications including methylation or acetylation [103,104]. Overall, DNA hypomethylation or histone acetylation are associated with transcriptional activation of regulatory genes that control development or hormone responses responsible for the totipotent state of the cells [39]. For example, in cotton, the level of CHH demethylation (one of the preferential DNA methylation contexts, with CG and CHG, where H can be an A, T or G) is a marker of somatic embryo differentiation [25]. By comparing SE recalcitrant *vs.* permissive genotypes, a hypermethylation of CHH is found, suggesting a repressive effect of methylation over SE-related genes expression, explaining why SE is less efficient for tissues with hypermethylated genomes [25]. Moreover, the DNA methylation level in callus decreases during embryogenesis and the important changes observed in the transcript levels during callus induction and somatic embryogenesis reflect the epigenetic reprogramming [39]. The use of deacetylation or demethylation inhibitors to promote the induction of somatic embryogenesis show that the chromatin profile of the tissue is a determining factor for SE induction, providing the evidence that chromatin regulations are directly involved in cell reprogramming and callus formation during SE [39,105,106,107].

POLYCOMB REPRESSIVE COMPLEXES (PRC) 1 and 2 are required for establishing and maintaining a stable epigenetic repression in response to developmental or environmental signals [108]. Briefly, PRC2 has a histone 3 lysine 27 trimethylation (H3K27me3) activity and PRC1 can recognize H3K27me3 and lead to chromatin compaction through histone H2A lysine ubiquitination (H2Aub) [49]. PCR2 is a main factor involved in cell identity to maintain a stable repression of embryogenesis program genes. PRC1 and PRC2 can repress the expression of several genes involved in cellular differentiation and callus development as well as somatic embryo formation including *WOX5*, *WOX8*, *AGL15*, *LEC1*, *LEC2*, *ABI3*, *FUS3*, *BBM*, *PIN1* or *PIN2* [109]. Consistent with these regulations, mutations in PRC2 subunits can lead to the formation of callus or abnormal development of vegetative tissues that resemble SE [110,111,112].

*PICKLE* genes (*PKL* and *PIKLE RELATED 1* and *2* – *PKR1* and *2*) are members of the CHD3 family of chromatin ATPases remodelers [113] and *VAL1* and *2* (*VIVIPAROUS/ABI3-LIKE*) are transcriptional regulators containing DNA and chromatin binding domains [114]. Both PKL and VAL have a repressive effect on *LAFL* genes expression during zygotic embryo development [49,113,114,115]. In *pkl* and *val1* mutants, the chromatin-based repression of *LAFL* is less important and consequently, the SE capacity is increased [52]. 

Mutations in genes involved in chromatin modifications, such as the DNA METHYLTRANSFERASE 1 (MET1) [116], KRYPTONITE (KYP) – an H2K9 METHYLTRANSFERASE [117], JUMONJI 14 (JMJ14) – an H3K4 DEMETHYLASE [118] and the HISTONE ACETYLTRANSFERASE 1 (HAC1) [119] lead to an alteration of *WUS* expression and a poorer formation of seedlings de novo after regeneration. Moreover, the functional analysis of *met1* showed that DNA methylation and histone modification control de novo regeneration through *WUS* expression and auxin signaling [120].

Among chromatin-modification proteins involved in SE processes AHL15, an Arabidopsis AT-hook binding domain transcription factor that is important for chromatin opening, induces SE in absence of auxin treatment when it is overexpressed [121]. AHL15 and its homologs are positively regulated during hormone-mediated induction of SE and are required for the induction through *BBM* overexpression. AHL15 has a role in the level of heterochromatin in somatic cells: in loss-of-function mutants, the amount of heterochromatin is higher in in vitro culture and the capacity of cells to form somatic embryos after 2, 4-D induction is altered. 

miRNAs are also important during plant development because they target regulatory genes including transcription factors and F-box proteins [122,123]. DICER-LIKE 1 (DCL1), is a RNase III-like enzyme responsible for the biosynthesis of most plant miRNAs [124]. The *dcl1* mutant, which is affected in the production of miRNAs, is not capable of inducing somatic embryogenesis [125]. This suggests that some miRNAs are crucial for SE induction processes, including auxin-related miRNAs that target genes of auxin perception, biosynthesis and signaling, for example: miR165/166, miR167, miR164, miR390 and miR393 [126].

In a recent study based on genome-wide analyses (ATAC-seq, ChIP-seq and RNA-seq), the authors observed a hierarchical organization of the transcriptional and chromatin mechanisms that regulate cellular reprogramming during plant somatic embryogenesis [127]. Their results suggest that the developmental stage of the explant used for tissue culture is at the top of the controlling hierarchy, as it determines the chromatin landscape. In fact, after germination, some specific chromatin permissive marks become inaccessible. This is the case for *AP2*, *B3* and *NF-Y* genes that encode the key TFs inducing somatic embryogenesis. After the developmental stage of the explant, the second level of this hierarchical organization is auxins that induce important changes in chromatin accessibility and, consequently in genetic expression of specific genes, especially AP2 and B3 transcription factors. Finally, the third level of the hierarchy is composed of the transcription factors themselves (AP2, B3 and NF-Y), which are expressed thanks to their opened chromatin status, and that initiate or regulate embryo formation.

## 6. Further Prospects

The mechanisms underlying the control of somatic embryogenesis are multiple, involve complex gene regulatory networks, hormonal and epigenetic controls, and remain poorly understood. For instance, *SOMATIC EMBRYOGENESIS RECEPTOR KINASE* (*SERK*) genes are leucine-rich repeat receptor-like kinase (LRR-RKK) [128] first identified in carrot as markers of embryogenic cells and involved in the transition from the vegetative to embryogenic state of the cells, but also in response to environmental signals and in plant development [129]. In vitro, SERK expression can be induced by various stresses such as drought, wounding, or by altering hormone balance in the culture media [17].

From a gene regulatory network point of view, somatic embryogenesis shares similarities with zygotic embryogenesis, and some differences. The transcriptome of embryonic cells is similar to those of zygotic embryos at the octant stage, but not earlier [21,130], and many stress-related genes are also detected in somatic embryos [131]. This emphasizes the similarity of molecular controls involved but also some specificities. Moreover, the similarity may result from a convergence of different morphogenic pathways [21]. The production of embryogenic cells in Arabidopsis callus needs the repression of biochemical pathways and root meristem genes, while activating gene networks involved in shoot patterning and polarized cell growth rather than inducing specific zygotic embryo networks [21].

It can be hypothesized that SE is controlled by embryogenic genes that acquired different or more specialized functions (neo- or sub-functionalization) or specific regulations during evolution that remain under specific environmental or physiological conditions capable of inducing embryogenesis. Different master regulatory genes of zygotic embryogenesis, such as *LAFL*, can be involved in the cellular stress necessary for transcriptional reprogramming. Moreover, the AP2 family of transcription factors, which contains AINTEGUMENTA-LIKE (ANT), BABY BOOM (BBM) and PLETHORA (APB), is involved in the maintenance of sporophytic meristems in Angiosperms [132]. Nevertheless, in ferns these genes are involved in the establishment of a gametophytic meristem without fertilization, and the overexpression of *ANT* or the *Brassica napus BBM* gene significantly increases the formation of sporophytes without gamete (apogamy), whereas the knockdown of *ANT* reduces this ability [133]. This type of conservation of an ancestral function, also suggested for several transcription factors genes [132], can explain the ability of Angiosperms to produce somatic embryos.

The production of somatic embryos appears to be strongly linked to specific cellular events. For further comprehension of these mechanisms, a better understanding of the gene networks involved is necessary at the cellular level: characterization of single cell transcriptome, epigenome, and proteome are required to develop better knowledge about molecular events in place at the cellular level. Recent technologies of single cell analyses or cellular transcriptome atlas are thus highly important to better appreciate the early gene regulatory networks associated with somatic embryogenesis. These techniques applied to samples enriched in embryonic cells with the help of specific marker genes or specific morphological markers build the basis of a better knowledge on early controls of somatic embryogenesis. For example, the use of *proLEC1* or *proLEC2* fused to a fluorescent protein marker is considered as a valuable marker to detect early events of SE production as used in [21]. Similarly, to monitor early molecular changes during SE, using *proBBM* should certainly be considered [52,70].

**Figure 3:**
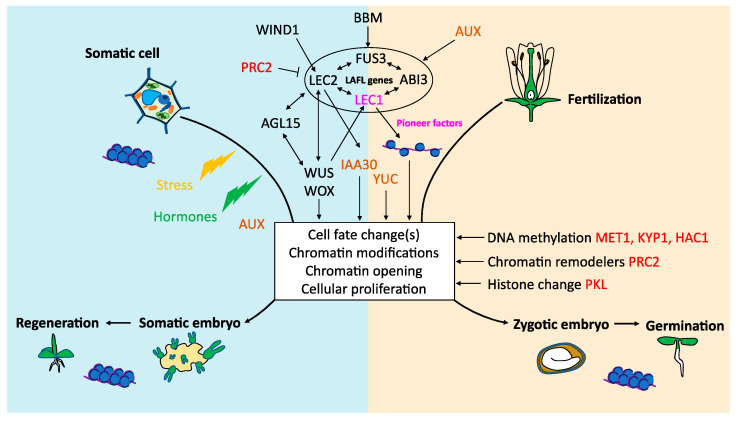
General overview of genes involved in somatic embryogenesis or zygotic embryogenesis in interaction with LEC1, ABI3, FUS3, and LEC2 (LAFL) transcription factors. Hormone-related genes are represented in brown, epigenetic regulators in red, transcription factors in black and pioneer factors in bright pink.

LEC1 is acting as a pioneer TF during embryogenesis to control *FLC* expression [134]. LEC2 forming some complexes with LEC1 can be involved in similar regulations. This pioneer activity may induce the key genes involved in the reprogramming of a cell to form a somatic embryo (Figure 3). Nevertheless, TFs such as LEC2, ABI3 or FUS3, which have several protein domains, can perform different roles (i.e., controlling early embryogenesis, reserve accumulation or desiccation tolerance), depending on their partners in different cellular environments (Figure 2 and Figure 3). Alternatively, the different functions can be linked if the ectopic triggering of the maturation processes induces stress in vegetative tissues leading to the initiation of somatic embryogenesis. 

## Data Availability

Not applicable.

## Abbreviations

ABA	Abscisic Acid
*ABI3*	*ABSCISIC ACID INSENSITIVE 3*
*AGL*	*AGAMOUS-LIKE*
*BBM*	*BABY BOO*M
*FUS3*	*FUSCA 3*
GA	Gibberellic Acid
*HAC1*	*HISTONE ACETYLTRANSFERASE 1*
*IAA30*	*INDOL-3-ACETIC ACID INDUCIBLE *30
*KYP1*	*KRYPTONITE 1*
*LEC1*	LEAFY COTYLEDON 1
*LEC2*	*LEAFY COTYLEDON 2*
*MET1*	*METHYLTRANSFERASE 1*
PGR	Plant Growth Regulators
*PKL*	*PICKLE*
*PRC2*	*POLYCOMB RESPONSE COMPLEX 2*
SE	Somatic Embryogenesis
*WUS*	*WUSCHEL*
ZE	Zygotic Embryogenesis
